# Identification of side- and shear-dependent microRNAs regulating porcine aortic valve pathogenesis

**DOI:** 10.1038/srep25397

**Published:** 2016-05-06

**Authors:** Swetha Rathan, Casey J. Ankeny, Sivakkumar Arjunon, Zannatul Ferdous, Sandeep Kumar, Joan Fernandez Esmerats, Jack M. Heath, Robert M. Nerem, Ajit P. Yoganathan, Hanjoong Jo

**Affiliations:** 1 School of Chemical and Biomolecular Engineering, Georgia Institute of Technology, Atlanta, GA, USA; 2 School of Biological and Health Systems Engineering, Arizona State University, Tempe, AZ, USA; 3 The Wallace H. Coulter Department of Biomedical Engineering, Georgia Institute of Technology and Emory University, Atlanta, GA, USA; 4 Mechanical, Aerospace and Biomedical Engineering, University of Tennessee Knoxville, TN, USA; 5 Parker H. Petit Institute for Bioengineering and Bioscience, Georgia Institute of Technology, Atlanta, GA, USA

## Abstract

Aortic valve (AV) calcification is an inflammation driven process that occurs preferentially in the fibrosa. To explore the underlying mechanisms, we investigated if key microRNAs (miRNA) in the AV are differentially expressed due to disturbed blood flow (oscillatory shear (OS)) experienced by the fibrosa compared to the ventricularis. To identify the miRNAs involved, endothelial-enriched RNA was isolated from either side of healthy porcine AVs for microarray analysis. Validation using qPCR confirmed significantly higher expression of 7 miRNAs (miR-100, -130a, -181a/b, -199a-3p, -199a-5p, and -214) in the fibrosa versus the ventricularis. Upon bioinformatics analysis, miR-214 was selected for further investigation using porcine AV leaflets in an *ex vivo* shear system. Fibrosa and ventricularis sides were exposed to either oscillatory or unidirectional pulsatile shear for 2 days and 3 & 7 days in regular and osteogenic media, respectively. Higher expression of miR-214, increased thickness of the fibrosa, and calcification was observed when the fibrosa was exposed to OS compared to the ventricularis. Silencing of miR-214 by anti-miR-214 in whole AV leaflets with the fibrosa exposed to OS significantly increased the protein expression of TGFβ1 and moderately increased collagen content but did not affect AV calcification. Thus, miR-214 is identified as a side- and shear-dependent miRNA that regulates key mechanosensitive gene in AV such as TGFβ1.

Calcific aortic valve (AV) disease is a slowly progressive disorder with a disease continuum that ranges from mild thickening of the valve to severe calcification with impaired leaflet motion. AV calcification is a strong risk factor for cardiovascular deaths and is a significant source of mortality worldwide^1^. Interestingly, explanted stenosed valves show signs of endothelial damage, inflammation, disrupted extracellular matrix (ECM), angiogenesis, and ossification on the fibrosa side of the valve with ventricularis side relatively unaffected^2^,^3^. Complex genetic programming as well as local hemodynamics that differ on either side of the valve could be factors in this side-dependent calcific valve disease^4^,^5^. Thus, understanding the basis for this increased susceptibility of fibrosa to inflammation can provide important clues about the regulatory mechanisms involved in AV calcification.

Shear stress is one such mechanical stimulus that greatly differs on either side of the valve, and plays an important role in this side-dependent disease^6^. Altered shear stresses can induce inflammatory markers such as VCAM-1, ICAM-1, TGFβ -1 BMP-4, which were significantly upregulated in fibrosa compared to ventricularis in endothelial and sub-endothelial regions^7^. Shear stresses are also known to regulate the expression of microRNAs (miRNAs)^5^,^8^,^9^. MiRNAs are emerging as potential master regulators as well as biomarkers for various cardiovascular diseases such as atherosclerosis, myocardial infarction, coronary artery disease, diabetes mellitus, hypertension, and aortic stenosis^10^. Shear stress is widely known to regulate miRNAs in vascular endothelial cells and atherosclerosis^11^,^12^, but the role of miRNAs in valvular endothelial cells is not well understood yet. Recently, shear dependent expression of miRNAs in human AV endothelial cells was demonstrated and discovered the miRNAs: miR-187, -214, -199a-5p, -181a, -181b, and -486-5p which could potentially regulate key cellular processes in AV disease progression^5^. Nigam, *et al.* has identified miRNAs that are differentially expressed between aortic stenosis and aortic insufficiency (miR-26a, -30b, -195) *in vivo* using whole, bicuspid valves and linked them to calcification-related genes, such as *Smad1/3*, *Runx2*, and *Bmp2* in AV interstitial cells *in vitro*^13^. Recently, Yanagawa *et al.* showed that miR-141a regulates the BMP-2 pathway as well and restored the valvular interstitial cell activation induced by TGF-β 1^14^.

Although there is a growing body of evidence suggesting the involvement of miRNAs in this side-dependent AV disease, the function of these miRNAs remains largely unknown. Lack of therapeutic treatments for AV calcification places an increasing demand on improving our current understanding of AV disease mechanisms. We aim to do so by identifying side- and shear-dependent miRNAs in AV endothelium. Understanding their function would uncover potential molecular mechanisms underlying AV disease, including secreted, circulating miRNAs that could act as potential biomarkers for AV disease.

Thus, the goal of this work is to: 1) determine the side-specific (fibrosa vs. ventricularis) miRNAs in the AV through microarray analysis and quantitative polymerase chain reaction (qPCR); and 2) investigate the shear dependency of a key side-dependent miRNA in AVs *ex vivo*; and 3) examine functional importance of a key shear- and side-dependent miRNA in AV disease.

## Results

### Side-dependent expression of miRNAs in fresh porcine AV determined by miRNA array analysis

**Figure 1:**
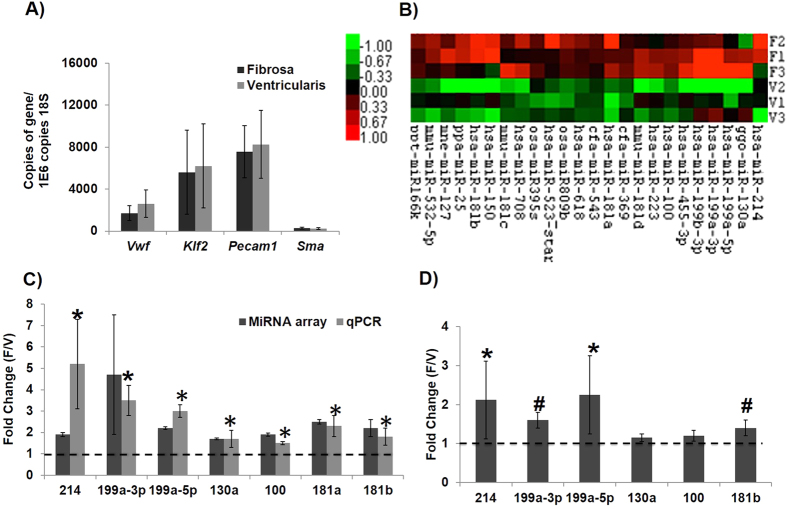
Differentially expressed miRNAs in porcine AV endothelium. (**A**) Endothelial-enriched RNA prepared from fibrosa and ventricularis of fresh porcine AVs showed robust expression of endothelial markers (*Vwf, Klf2*, and *Pecam1*), but not an interstitial cell marker (*Sma*). This endothelial-enriched RNA was used for both microarray analysis and validation of selected miRNAs using qPCR. n =  9. (**B**) Heat map of differentially expressed miRNAs in porcine AVs generated using significance of microarray, SAM analysis. F: fibrosa, V: ventricularis. Red shows increased expression and green shows decreased expression. n =  3. (**C**) Of 24 miRNAs, 7 side-dependent miRNAs were confirmed by qPCR at total level (pre + mature). n =  3. (**D**) Four side-dependent miRNAs were also confirmed at mature miRNA level. n =  7. Three samples were pooled/isolation for each n in A, B, C and D. *p <  0.05, ^#^p <  0.1. Data was tested for normality by Anderson-Darling test and one-way ANOVA with Tukey’s post-hoc test was done to statistically analyze the data.

Endothelial-enriched total RNAs were isolated from each side (fibrosa vs. ventricularis) of fresh porcine AV and were used to examine its endothelial RNA purity. As shown in the Fig. 1A, these endothelial-enriched RNA preparations showed abundant expression of the endothelial cell markers (*vWF*, *Klf2 and Pecam1*) with a very low level of a valve interstitial cell (VIC) marker (*Sma*), n =  9 (RNA for each n for fibrosa or ventricularis was pooled from 3 to 4 AV leaflets). These results show a clean preparation of endothelial-enriched RNA from porcine AV endothelium with minimal contamination of VICs. Upon purity validation, each side-specific endothelial RNAs obtained from each pig was pooled (3 to 4 per pooled samples) for miRNA array analysis (n =  3 fibrosa and ventricularis each) using Affymetrix Multispecies Microarray Gene Chips (since porcine-specific Chips were not available) and validation by qPCR.

### Generation, Analysis, and Validation of miRNA Arrays from the Side-specific, Endothelial-enriched Total RNA

The side-specific miRNA microarray data was analyzed using Significance of Microarray Analysis with 25% false discovery rate. From this, we found 24 side-dependent miRNAs. Figure 1B shows a heat map representation of these side-dependent (fibrosa vs. ventricularis) miRNAs (n =  3 each). Interestingly, all side-dependent miRNAs detected in this analysis showed higher expression in the fibrosa relative to the ventricularis. A complete list of changed miRNAs is listed in the Supplementary Data online. Based on detection level and consistency in multiple samples, we were able to validate only 7 of these 24 miRNAs by an independent assay using qPCR. As shown in Fig. 1C, the 7 miRNAs were confirmed by qPCR at total (mature and pre-miR forms) miRNA level. These seven miRNAs are miR-100, -130a, -181a/181b, -199a-5p, -199a-3p, and -214. Interestingly, miR-199a and miR-214 are expressed as miR-199a~214 cluster^15^. Further, as shown in Fig. 1D, four out of seven miRNAs (miR-181a, -199a-5p, -199a-3p, -214) were further confirmed by qPCR at the mature miRNA level as well. Moreover, as per miRBase^16^, these side-dependent miRNAs: miR-100, miR-199a-3p, miR-199a-5p, miR-130a, miR-181a, miR-181b, and miR-214 are homologous with miRNAs in human: miR-100, miR-199a-3p, miR-199a-5p, miR-130a-3p, miR-181a-5p, miR-181b-3p, and miR-214-3p, respectively.

### *Ex vivo* shear stress system and AV calcification model

**Figure 2:**
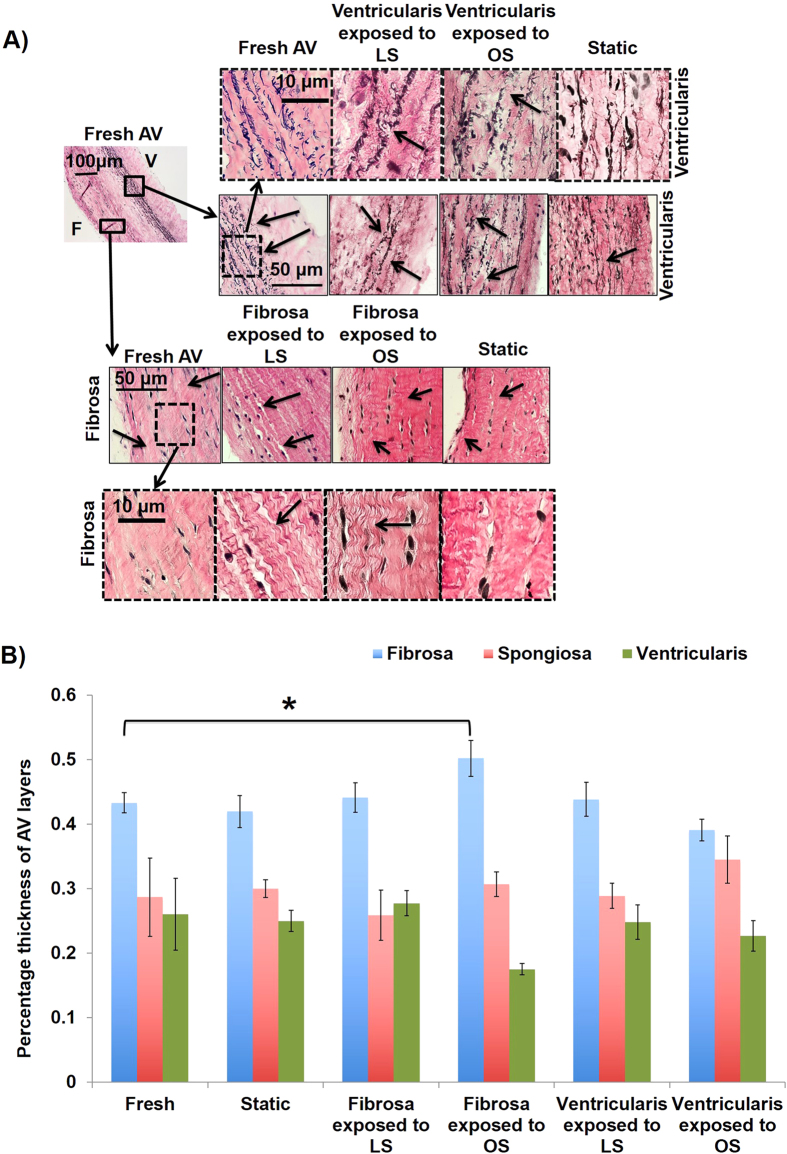
OS induces most changes in the fibrosa. (**A**) When either fibrosa or ventricularis was exposed to OS or LS for 2 days, histological changes (relative to fresh and static culture) in collagen and elastin fiber distribution were observed. F: fibrosa, V: ventricularis. (**B**) Quantification of images showed that OS significantly increased the thickness of the fibrosa compared to the fresh AVs (*p <  0.05), while the thickness of the ventricularis and the spongiosa remained unaltered. n =  4. The data was not normally distributed so a non-parametric analysis (Mann-Whitney test) was done to statistically analyze the data.

**Figure 3:**
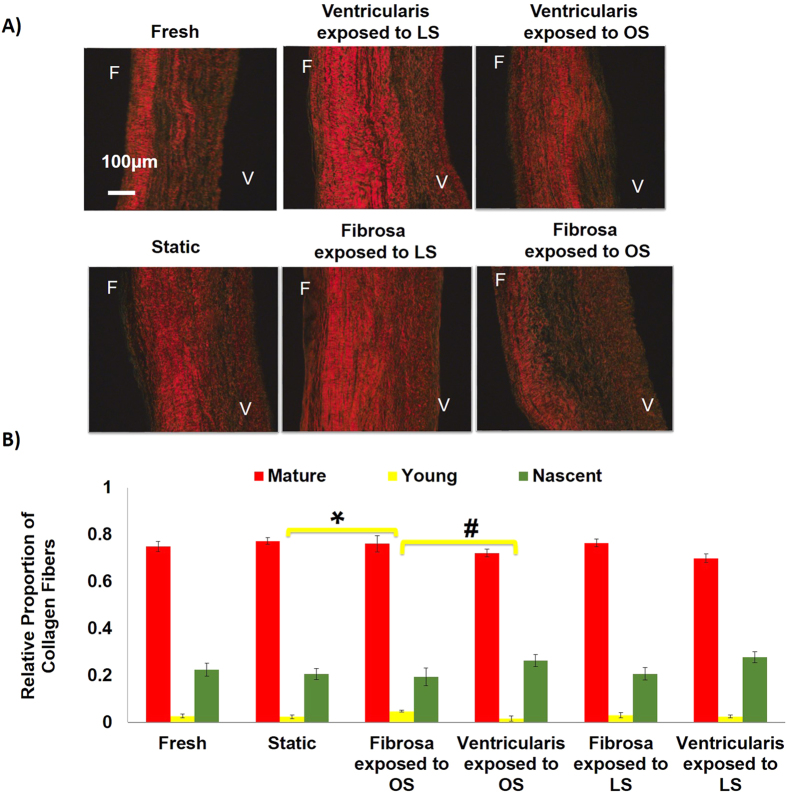
OS induces most changes in collagen fiber content in fibrosa. (**A**) Picrosirius red staining showing the distribution of mature, young and nascent collagen fibers in fibrosa or ventricularis exposed to OS or LS for 2 days (mature: red, young: yellow, nascent: green). F: fibrosa, V: ventricularis. (**B**) Quantification of relative proportions of collagen fibers showed that most changes were observed in fibrosa when exposed to OS. Specifically, young collagen fiber content increased in fibrosa exposed to OS compared to ventricularis exposed to OS (1.95 fold, ^#^p =  0.09) and statically cultured AVs (1.04 fold, *p =  0.041). The mature and nascent collagen fiber content was preserved in all culture conditions. n =  6. The data was not normally distributed so a non-parametric analysis (Mann-Whitney test) was done to statistically analyze the data.

**Figure 4:**
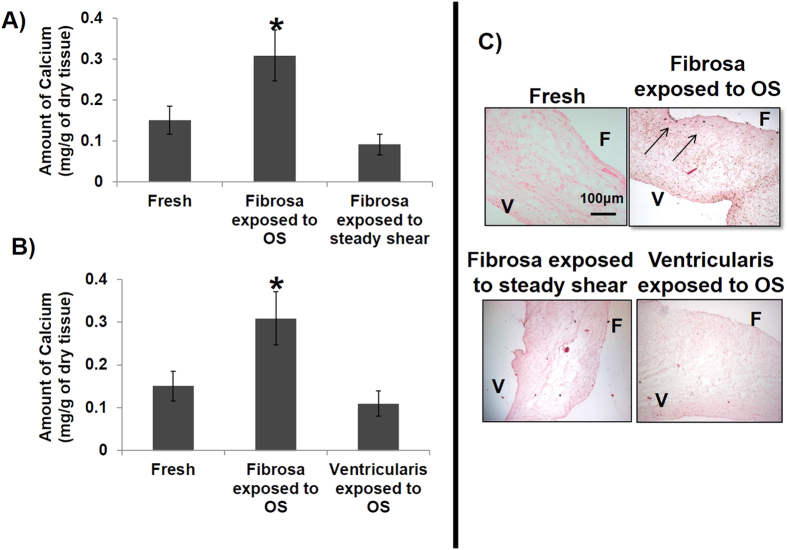
Oscillatory nature (+/−) of shear stress induces calcification in fibrosa. (**A**) The calcium level was significantly higher in fibrosa exposed to oscillatory shear, OS (± 5 dyne/cm^2^) compared to steady shear (5 dyne/cm^2^) for 3 days. n =  8, *p <  0.05. (**B**) The amount of calcium induced in the fibrosa when exposed to OS was significantly higher than that in ventricularis and fresh AVs. n =  8, *p <  0.05. (**C**) Consistent with the quantitative result, calcium nodules were observed only in the fibrosa compared to all other conditions. Calcium nodules appeared black (arrows). n =  6. F: fibrosa, V: ventricularis. Data was tested for normality by Anderson-Darling test and one-way ANOVA with Tukey’s post-hoc test was done to statistically analyze the data.

Next, we decided to determine the functional importance of the side-dependent miRNAs in AV calcification in an *ex vivo* shear stress system. First, we tested whether AV leaflets can be calcified by either unidirectional pulsatile laminar shear stress (LS) or oscillatory shear (OS) using the *ex vivo* shear system^7^ (also refer to methods for additional details on shear conditions). Within 2 days of culture in regular media, the overall tri-layered (fibrosa, spongiosa and ventricularis) composition of AV ECM was preserved under all shear conditions. As shown in Fig. 2A, fiber architecture of collagen and elastin was preserved when fibrosa and ventricularis were exposed to their respective shear stress conditions (fibrosa exposed to OS and ventricularis exposed to LS). Fragmentation of elastin fibers and loss of collagen crimping was observed in ventricularis and fibrosa, respectively, when the shear stress profile was altered (fibrosa exposed to LS, ventricularis exposed to OS). However, the thickness of the fibrosa and its collagen fiber composition varied significantly only when the fibrosa was exposed to OS compared to all other shear conditions (Figs 2B and 3A,B). Next, the effect of OS on AV calcification was tested. As shown in the Fig. 4A, calcification in the fibrosa side was significantly higher when exposed to the bi-directional OS (± 5 dyne/cm^2^ at 1Hz), but not uni-directional steady shear of the same magnitude (5 dyne/cm^2^). This suggests that the OS condition triggers calcification in the fibrosa. Further, this response proved to be side-dependent, since no calcification was observed when the ventricularis was exposed to OS (Fig. 4B). Consistent with this quantitative result, the Von Kossa mineralization stain (Fig. 4C) also showed localization of black calcium nodules in the fibrosa side exposed to OS but not in any other conditions. In these calcification studies, TUNEL staining showed no visible apoptosis in either the fibrosa or the ventricularis when exposed to OS (see Supplementary Fig. S2).

### Shear stress and miR-214 in AV leaflets *ex vivo*

Next, we studied the role of the side-dependent miRNAs in AV inflammation and calcification using the *ex vivo* shear and calcification model. To decide which of the 7 side-dependent miRNAs should be studied in the inflammation and calcification study, we carried out a literature survey. Based on this, we identified miR-214 to be involved in proliferation^17^,^18^, cell death^19^,^20^,^21^, cell cycle^17^, migration^19^,^22^, fibrosis^23^, and inflammation^18^,^20^,^21^, processes that are well known to lead to AV calcification. Further, the miR-214 has also been associated with the severity of coronary artery disease^24^ and osteogenic differentiation^25^. Hence, we chose to further investigate the functional role of this side-dependent miRNA, miR-214 in AV pathophysiology. We first examined whether the expression of miR-214 was regulated in a side- and shear-dependent manner by exposing AV leaflets (fibrosa or ventricularis) to OS or LS *ex vivo*. Also, since the shear stress affects both the endothelial and interstitial cells as well as the technical difficulty of obtaining clean endothelial-enriched RNA preparation following shear treatment, the expression of miR-214 in shear stress studies was assessed in the total tissue containing both endothelial and interstitial cells rather than the endothelial cells alone.

**Figure 5:**
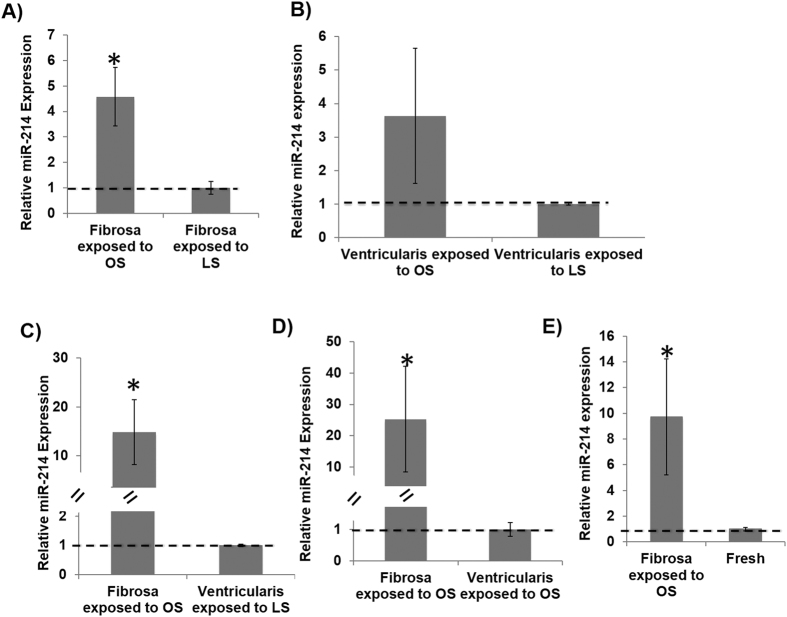
miR-214 is side-as well as shear-dependent in fibrosa. (**A–D**) Fibrosa-side or ventricularis-side of AV leaflets were exposed to OS vs. LS for 2 days, and total RNAs were prepared from the sheared leaflets for miR-214 analysis by qPCR. (**E**) Exposure of fibrosa to OS also increased miR-214 expression in the sheared leaflets compared to fresh AV. *p <  0.05. n =  6, pooled 3 samples/isolation. Data was tested for normality by Anderson-Darling test and one-way ANOVA with Tukey’s post-hoc test was done to statistically analyze the data.

As shown in Fig. 5A, expression of miR-214 was upregulated by OS compared to LS in fibrosa. Interestingly, this OS effect was not statistically significant (p >  0.05) in ventricularis (Fig. 5B). When compared to ventricularis (LS or OS), fibrosa under OS showed significantly higher miR-214 expression (Fig. 5C,D). Also, the expression of miR-214 in fibrosa was significantly increased by OS compared to fresh AV (Fig. 5E). This result indicates that miR-214 is regulated in a shear- and side-dependent manner in fibrosa, but not in ventricularis, consistent with the above array data using fresh porcine AV leaflets.

### Shear stress and identification of potential mRNA targets of miR-214 in AV leaflets *ex vivo*

**Figure 6:**
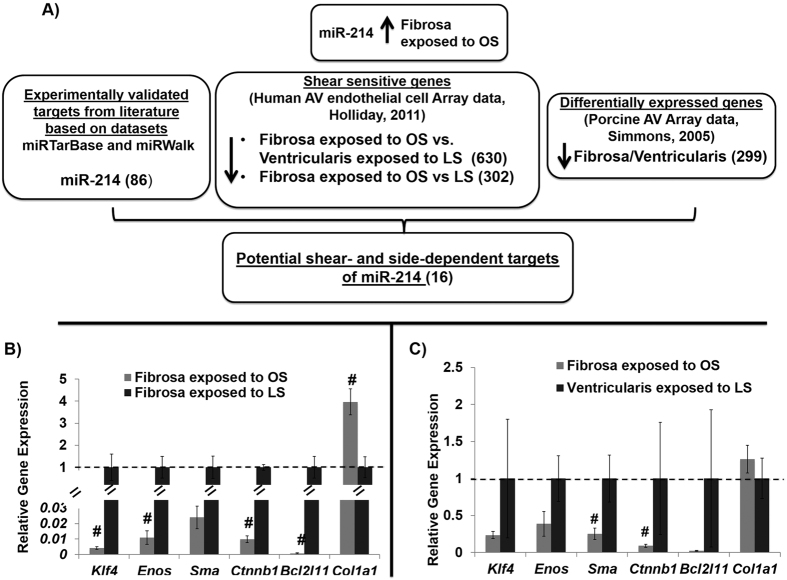
Side- and shear- dependent potential targets of miR-214. (**A**) Using this bioinformatics analysis and target filtration scheme, sixteen potential shear- and side-dependent targets *was* filtered for miR-214. (**B,C**) Overall 5 out of 6 targets, *Klf4*, *Enos*, *Ctnnb1*, *Bcl2l11* and *Sma* showed an inverse relationship with miR-214 expression in the AV leaflets when their fibrosa or ventricularis-side was exposed to OS for 2 days. n =  3 (pooled 3 samples/isolation), ^#^p ≤  0.1. The data was not normally distributed so a non-parametric analysis (Mann-Whitney test) was done to statistically analyze the data.

Next, we determined gene targets of miR-214 in AV by combining 1) predicted genes according to miRTarBase^26^ and miRWalk^27^, 2) shear-sensitive genes identified in human AV endothelial cell by gene array^5^, and 3) side-dependent genes identified in porcine AV fibrosa vs. ventricularis^28^, as outlined in Fig. 6A (see methods for additional details on this bioinformatics analysis). Using this strategy, 16 out of 86 potential mRNA targets of miR-214 were filtered. To establish a link between miR-214, its target genes and AV pathology, we chose to focus on six targets (from the filtered list of 16 targets) for further investigation based on their potential importance in AV pathophysiology. These are *Enos*^29^, *Klf4*^30^,^31^*, Sma*^32^, *Ctnnb1*^33^,^34^, *Bcl2l11*^35^, and *Col1a1*^36^.

Of these 6 potential targets, four (*Klf4, Enos*, *Ctnnb1*, *Bcl2l1)* showed a down-regulation trend (p ≤  0.1) in fibrosa when exposed to OS compared to LS (Fig. 6B). On the other hand, only *Sma* and *Ctnnb1* showed a downregulation trend (p ≤  0.1) in fibrosa exposed to OS when compared to ventricularis exposed to LS (Fig. 6C, also see Supplementary Fig. S3). In contrast, *Col1a1* expression tended to increase by OS in fibrosa, suggesting that this gene may not be a shear-dependent target of miR-214 in AV. So, 5 out of 6 potential targets (*Klf4, Enos*, *Ctnnb1*, *Bcl2l1 and Sma,* but not *col1a1)* were chosen for further investigation.

### Silencing of miR-214 in AV leaflets *ex vivo*

**Figure 7:**
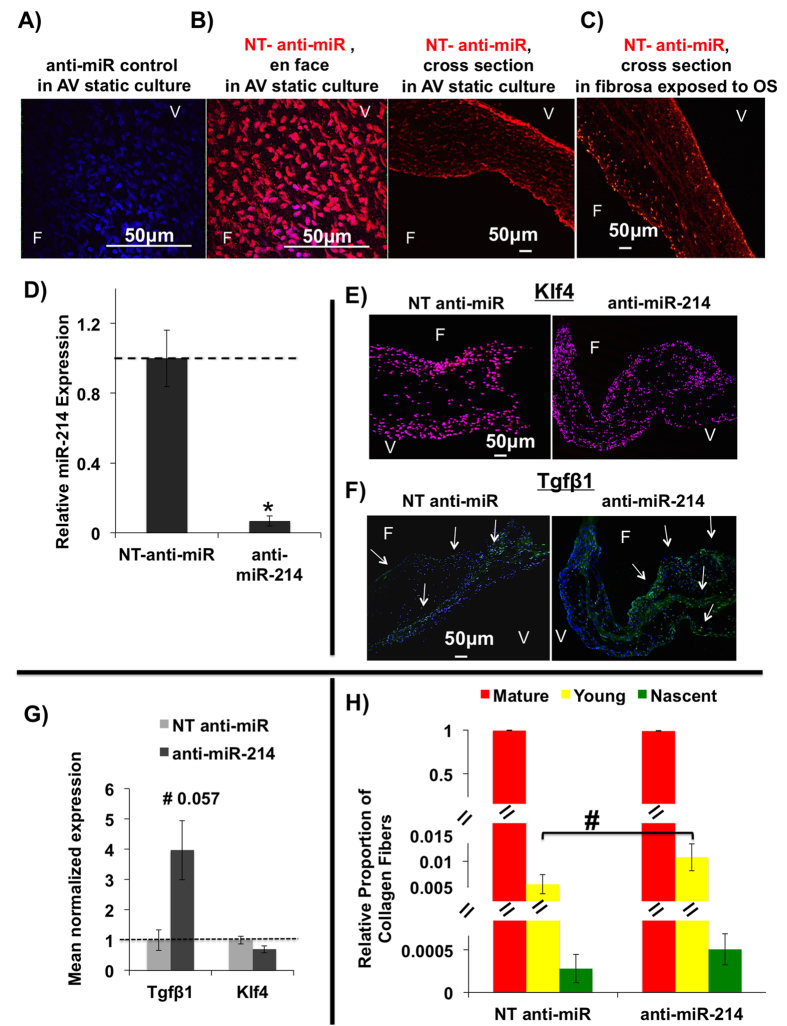
miR-214 targets TGFβ1 in whole AV leaflets when the fibrosa is exposed to OS. (**A**,**B**) Compared to no anti-miR control (**A**), Texas 615-labeled non-targeting (NT) anti-miR (red) showed transfection in both valvular endothelial and interstitial cells of the statically cultured AV (**B**). Nuclei counterstained with DAPI. (**C**) Tex615-labeled NT anti-miR (red) was transfected and retained in the tissue even after exposing the fibrosa to OS. F: fibrosa, V: ventricularis. (**D**) The miR-214 was significantly silenced using anti-miR-214 (400 nM) in the whole AV leaflet following OS exposure to fibrosa. *p =  0.004. n =  5 (pooled 3 samples/isolation). (**E,F**) Silencing of miR-214 did not appear to alter the protein expression of Klf4 (Rhodamine Red X) (**E**) but increased the protein expression of TGFβ 1 (DyLight 488) (**F**) as shown by the immunostaining. Expression of Klf4 was seen through out the tissue in both fibrosa and ventricularis sides, while expression of TGFβ 1 was seen predominantly in the fibrosa side compared to the ventricularis. (**G**) Quantification of the immunostaining images (**E,F**) showed that while silencing of miR-214 significantly increased the expression of TGFβ 1 (p =  0.057, n =  3), it did not alter the expression of Klf4. (**H**) Quantification of relative proportions of collagen fibers showed no statistically significant change in young collagen fiber content in fibrosa when miR-214 was knocked down (0.94 fold, ^#^p =  0.09) n =  4.

To validate whether miR-214 indeed downregulate those potential target genes and to determine the functional role of miR-214 in AV pathophysiology, we developed a miRNA silencing protocol. The transfection of fluorescence (Texas Red615)-labeled non-targeting (NT) anti-miR to whole AV leaflets in static cultures compared to the no anti-miR control group was observed in both endothelial and interstitial cells, as shown in Fig. 7A,B. Also, the transfection of Tex615-labeled NT anti-miR was observed throughout the sheared tissue (fibrosa exposed to OS) (Fig. 7C). This further confirmed that the anti-miR was delivered to both the endothelial and interstitial cells of AV and that the shear stress retained the anti-miR in the tissue. Following the same protocol, the miR-214 was also significantly silenced in fibrosa exposed to OS, as shown in Fig. 7D. As a control, we examined whether the use of anti-miR caused apoptosis in the AV tissue, but it did not (see Supplementary Fig. S2).

### Shear stress dependent functional role of miR-214 in AV disease *ex vivo*

Following the silencing of miR-214 in whole AV leaflet tissue with anti-miR-214, the fibrosa was exposed to OS and the expression of its potential gene targets identified above was determined using qPCR. Out of 5 targets tested (*Klf4, Enos*, *Ctnnb1*, *Bcl2l1 and Sma)*, only the expression of *Klf4* was significantly upregulated (p =  0.05), while the changes in *Sma* showed a trend (p =  0.11) without reaching a statistical significance (see Supplementary Fig. S4). This suggests that *Klf4* could be a potential target of miR-214 in AV. To confirm if Klf4 is a target gene, we then examined its protein expression using immunofluorescence staining. As seen in Fig. 7E, the expression of klf4 (Rhodamine Red X) was observed throughout the AV leaflet tissue, in both endothelial and interstitial cells. However, quantification of immunostaining (Fig. 7G) showed that silencing of miR-214 did not significantly increase the expression of Klf4. This suggests that miR-214 regulates the gene expression of Klf4, but that does not translate at the protein level.

Using immunofluorescence staining, we next tested whether silencing miR-214 affects the expression of TGFβ 1, a key cytokine involved in inflammatory and osteogenic pathways that lead to AV calcification. As seen in Fig. 7F, in both control and anti-miR-214 groups, the protein expression of TGFβ 1 (DyLight 488) was observed in endothelium and sub endothelium as well as in the interstitial cells, with predominantly higher expression in the fibrosa side compared to the ventricularis. Also, higher expression of TGFβ 1 was observed after miR-214 was silenced using anti-miR-214 compared to the non-targeting anti-miR control group. Quantification of the same (Fig. 7G) showed that silencing of miR-214 significantly increased the expression of TGFβ 1 (p =  0.057) compared to the non-targeting anti-miR control group, suggesting that miR-214 may be regulating the expression of TGFβ 1, a key player in AV pathogenesis^37^. We also investigated if silencing miR-214 altered the collagen composition of sheared AV leaflets. Silencing miR-214 also moderately increased young collagen fiber content but was not statistically significant (p = 0.09) (Fig. 7H).

**Figure 8:**
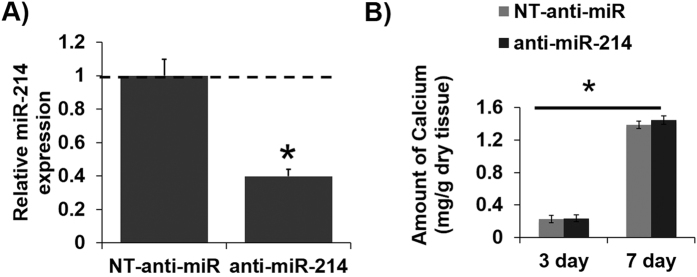
Silencing of miR-214 did not alter calcification in whole AV leaflets. (**A**) The miR-214 was significantly silenced using anti-miR-214 (400 nM) in AV leaflets with the fibrosa exposed to OS in osteogenic media (7 day culture). *p <  00.05. n =  6 (pooled 3 samples/isolation). (**B**) Silencing of miR-214 did not change the calcium levels induced by exposure of the fibrosa to OS compared to the NT anti-miR even after 7 day culture. However increase in culture duration (3 day to 7 day) increased the calcium levels in both NT-anti-miR and anti-miR-214 conditions. n =  6. P <  0.05.

Given its significant effect on increasing TGFβ 1, we next tested whether anti-miR-214 would inhibit OS-induced AV calcification. However, anti-miR-214 did not show any effect on calcification induced by exposure of fibrosa with OS compared to the NT anti-miR control group even after 7 days of culture (Fig. 8A,B). This suggests that miR-214 possibly modulates the genes in early stages of AV pathogenesis rather than the later stages (calcification).

## Discussion

The major findings of this work are: 1) identification of differentially expressed miRNAs on either sides of the AV endothelium; 2) development of an *ex vivo* shear system to be able to investigate the shear dependency and functional role of these miRNAs; 3) identification of possible targets of a key side- and shear-dependent miRNA, miR-214; and 4) shear-dependent functional role of miR-214 in AV by knocking down its expression. Most notably, our results suggest that while the shear- and side-dependent miR-214 targets TGFβ 1 in AV, its rescue by anti-miR-214 was not sufficient to affect OS–mediated AV calcification.

Native AV leaflets express inflammation and calcification markers in the fibrosa making it predisposed to disease^28^. To further understand this side-dependent gene expression pattern and its role in the calcific AV disease process, we investigated the expression of miRNAs that are differentially expressed on either sides of healthy valvular endothelium. Since the miRNAs are critical regulators of gene expression, they could play an important role in AV disease as well. For this purpose, we were able to successfully isolate side-dependent endothelial RNA from healthy porcine AV leaflets, which abundantly expressed endothelial cell markers (*Pecam1, vWF, Klf2*) and little interstitial cell marker (*Sma)*, suggesting minimal contamination of RNA from interstitial cells. Of the 24 miRNAs detected by the side-specific porcine AV endothelial array, we confirmed 7 miRNAs at total (both pre- and mature forms) level and further confirmed 4 miRNAs (miR-199a-5p, -214, -199a-5p and-181b) at mature miRNA level. Interestingly, all side- dependent miRNAs detected were higher in the fibrosa, confirming the differential gene expression pattern on either sides of AV. To our knowledge, this is the first study to report the differential expression of miRNAs in healthy AV tissues, and thus the importance of these miRNAs in AV disease should be determined. Of the miRNAs validated, as discussed earlier, several had been studied in other contexts, such as cancer, fibrosis, myocardial infarction etc. These miRNAs also play an important role in cellular functions known to be important in AV disease including apoptosis, migration, proliferation, endothelial-to-mesenchymal transition, inflammation, and calcification. Based on this classification, miR-214 was found to be involved in all of these pathways (proliferation^17^,^18^, cell death^19^,^20^,^21^, cell cycle^17^, migration^19^,^22^, fibrosis^23^, and inflammation^18^,^20^,^21^) and its expression has also been linked to the severity of coronary artery disease^24^. Hence, we chose to further investigate the functional role of this side-dependent miRNA, miR-214 in AV pathophysiology.

Calcific lesions occur predominantly in the fibrosa side of the AV, making it a side-specific disease^38^,^39^. Differing shear stress profiles on either side of the valve plays a potential role in this side-dependent AV disease^40^. So an *ex vivo* shear system was used to develop a model system that can induce both early (gene expression, ECM, inflammation) as well as late (calcification) stages of AV disease in a side-dependent manner. Increased thickness of fibrosa and alterations in collagen fiber composition in response to OS within 2 days suggests that fibrosa is quite sensitive to changes in its mechanical environment and responds by altering its matrix architecture. ECM on ventricularis side, when exposed to LS, was relatively unaffected, as expected. Further, changes in ECM constitute active remodeling that can affect the valve cell phenotype^41^, dictate the miRNA expression^42^, and gene expression changes and vice versa^34^,^43^. As expected the OS also induced calcification only in the fibrosa but not the ventricularis and this calcification required the oscillatory nature of the shear stress. Thus, the OS induced most changes in the AV ECM, increased the thickness of the fibrosa, and induced calcification in the fibrosa but not the ventricularis.

The OS also increased expression of miR-214 in fibrosa compared to ventricularis and freshly isolated AV tissues. The lower expression of miR-214 in ventricularis was consistently maintained regardless of shear stress, suggesting that miR-214 might not be shear-sensitive in ventricularis. Although the increased expression of miR-214 in the fibrosa is consistent with our microarray results (Fig. 1C,D), it is possible that this increased miR-214 expression in response to OS could either be an adverse or a protective response. A detailed bioinformatics analysis showed that miR-214 potentially regulates genes that play a role in important cellular processes. Six of these potential target genes (*Enos*^29^, *Klf4*^30^,^31^, *Sma*^32^, *Ctnnb1*^33^,^34^, *Bcl2l11*^35^, and *Col1a1*^36^) that are well known for their involvement in AV pathogenesis, were chosen for further investigation. This focused approach was used to determine a link between miR-214, potential target genes and AV pathogenesis. Interestingly, the expression of 5 of 6 potential mRNA targets of miR-214 (except for *Col1a1*) was downregulated in fibrosa when exposed to OS, and thus, inversely correlated with the increased expression of miR-214. Particularly, the OS decreased the expression of anti-inflammatory genes *Enos and Klf4* in the fibrosa, making fibrosa more susceptible to disease. Also changes in valve cell phenotype (*Sma),* collagen fibers, increased collagen type-1 (*Col1a1*) combined with increased thickness of fibrosa (all being the indicators of valve ECM remodeling), were noticed only in fibrosa in response to OS but not LS. These changes observed in the ECM composition and its related genes can also potentially affect the miRNA expression and vice versa^42^. Thus, OS can create a pro-inflammatory environment and pro-sclerotic environment, preferentially in the fibrosa side^6^,^7^,^44^, that may be correlated with the higher expression of miR-214 observed in fibrosa.

Successful transfection of anti-miR to AV endothelial and interstitial cells in sheared AV leaflets was demonstrated without compromising cell viability. The increased expression of miR-214 in response to OS in the fibrosa was also successfully silenced using anti-miR-214. We then performed a systematic study to determine the role of miR-214 in AV pathogenesis. We first identified the target genes regulated by miR-214 by silencing miR-214 using anti-miR-214. Out of 5 potential targets tested, one anti-inflammatory gene, *Klf4* appeared to be regulated at the gene level but not at protein level (Fig. 7G). However, anti-miR-214 showed no effect on *Sma*, another key EndoMT marker (see Supplementary Fig. S4). On the other hand, miR-214 silencing significantly increased the protein expression of TGFβ 1 indicating that miR-214 may be involved in the pathways regulated by TGFβ 1 such as inflammation, fibrosis and endothelial-to-mesenchymal transition (Endo-MT)^37^,^45^,^46^. Perhaps, in this mixed population study (involving both valvular interstitial and endothelial cells), the SMA and TGFβ 1 do not trend similarly as expected^32^. It is also possible that the expression of SMA is regulated by miRNAs other than miR-214^47^. On the other hand, the observed moderate increase in young collagen content (Fig. 7H) in the presence of anti-miR-214 could be mediated by TGF-β 1^48^. One hypothesis could be that TGFβ 1 induced by OS can accelerate the AV fibrosis, by activating the differentiation of quiescent VICs and endothelial cells into osteoblast like phenotype^34^. So the increased expression of miR-214 in fibrosa when exposed to OS could be a protective response to regulate the TGFβ 1 induced pro-inflammatory and pro-calcific events. Although miR-214 regulates the TGFβ 1, one of the key cytokines involved in AV calcification, unfortunately, silencing of miR-214 did not affect the calcification induced by OS (Fig. 8A,B). It is also possible that miR-214 may regulate calcification via a different mechanism or there might be other miRNAs involved.

The mixed results we observed in this study can be attributed to the combined responses of both valvular endothelial and interstitial cells, as opposed to the endothelial cells alone. While we were able to collect clean endothelial-enriched RNA from fresh AV leaflets, it was challenging to isolate endothelial-enriched RNA once AV leaflets were sheared for more than two days. This made it difficult to determine the effects of anti-miR-214 on its gene targets by qPCR in AV endothelium *ex vivo*. As such, in this study, anti-miR-214 did not show any significant effect on AV calcification and remodeling. This may be partly due to: 1) anti-miR-214 was added to the whole AV leaflets, therefore it would affect many other gene targets some of which may have opposite effects than its known target TGFβ 1 cancelling out anti- vs. pro-calcific events; and 2) it may require a much longer time period to observe the effect of anti-miR-214 on AV calcification. Given the side-specific expression of miR-214 in the fibrosa endothelium, these results suggest a complex role of miR-214 and that it may work in concert with other miRNAs to regulate the AV pathophysiology.

On the other hand, the expression of miR-214 and its target genes can also be influenced by other mechanical stimuli such as cyclic strain, stretch, bending, and pressure etc., experienced by AV^49^. Also, the calcification induced by elevated cyclic stretch appears to be significantly higher^50^ compared to that of shear stress, as observed in this study (Fig. 4A–C). Since miR-214 did not have an effect on OS-induced calcification in AV, in future studies, it will be worthwhile to investigate its role in stretch-mediated calcification.

We have shown that miR-214 is a shear- and side- dependent miRNA that plays an important role in AV endothelium. Further, the oscillatory shear stress induced a higher expression of miR-214, increased thickness of the fibrosa and collagen content, altered the ECM, and created a pro-fibrotic environment via a miR-214 - TGFβ 1 dependent pathway. Given the critical role for TGFβ 1 in valvular pathophysiology, we thus speculate that miR-214 could be a key miRNA involved in AV pathogenesis in a side- and shear- dependent manner. Further research is warranted to understand the complex functional role of miR-214 in mediating AV inflammation and calcification.

## Materials and Methods

### Porcine Aortic Valve Tissue Harvest

Hearts were obtained from healthy, female, non-pregnant pigs (aged 6 to 12 months) immediately after slaughter from a local abattoir (Holifield Farms, Covington, GA). The AV leaflets were immediately excised and thoroughly rinsed in sterile nuclease-free Dulbecco’s phosphate buffered solution (dPBS; Sigma-Aldrich, MO) at 4 °C. These excised leaflets were either placed in RNAlater (Qiagen, CA; for generation of miRNA array samples) or nuclease-free dPBS at 4 °C (shear stress studies, and miRNA silencing studies). Valves were placed on ice and transported back to the laboratory. Upon arrival in the laboratory, dissected AVs were transferred to sterile dPBS at 4 °C under the sterile laminar flow hood and processed accordingly.

### Isolation and purity assessment of Endothelial-enriched Total RNA from Porcine Aortic Valve leaflets for Microarray Studies

To isolate endothelial enriched total RNA for microarray studies, the freshly isolated AV leaflets saved in RNAlater (from local abattoir) were used. After thoroughly rinsing with sterile nuclease-free dPBS under the sterile laminar flow hood, valves were then arranged on thin pieces of plastic so that the fibrosa (or ventricularis) sides were facing up. The plastic was slightly cupped and then 200 μ L of Qiazol was poured over one side of the leaflet for about 10 s, and finally collected into a micro centrifuge tube. The leaflet was then washed well with sterile nuclease-free dPBS three times and the same method was repeated using a new piece of plastic on the opposite side of the valvular leaflet. To obtain RNA yield to the required level for microarrays and qPCR analysis, total RNA from three to four porcine AVs was pooled side-specifically (fibrosa and ventricularis). The miRNeasy kit (Qiagen, CA) was used for RNA isolation and purification.

To assess for endothelial purity in samples before submitting for microarray analysis, qPCR for three endothelial cell markers, von Willebrand factor (*Vwf*), platelet-endothelial cell adhesion molecule-1 (*Pecam1*) and Kruppel-like factor 2 (*Klf2*), as well as one vascular interstitial cell marker, smooth muscle alpha actin (*Sma*), was conducted n =  9 (RNA for each n for fibrosa or ventricularis was pooled from 3 to 4 AV leaflets). Additional quality control to investigate the integrity of the RNA samples was completed by the Emory Biomarker Center, including Taqman analysis for RNU24 (an abundant small nucleolar RNA) and Bioanalyzer analysis.

### Generation, Analysis, and Validation of microRNA Arrays from the Side-specific Endothelial-enriched Total RNA

Endothelial-enriched RNA, isolated as described above, was submitted to the Emory Biomarker Center for analysis. The total RNA (n =  3 fibrosa and ventricularis each) was then hybridized on Affymetrix Multispecies Microarray Gene Chips and fluorescence intensity values were determined for analysis. We used this human microarray chip due to the unavailability of a porcine microarray. All samples used in miRNA microarray (n =  3, pooled samples each for fibrosa and ventricularis) passed quality control as well as appropriate levels of RNU24, an abundant housekeeping small nucleolar RNA used as an internal control (data not shown). The side-specific miRNA microarray data was then analyzed using Significance of Microarray Analysis (Stanford). A false discovery rate (FDR) of 25% was observed. A heat map was then generated using Cluster 3.0 and Treeview. Selected abundant miRNAs were validated via qPCR using Ncode kit (Invitrogen, NY) and SYBR green (Applied Biosystems, NY). Primers specific for these miRNAs were obtained from Integrated DNA Technologies (CA).

### Bioinformatics analysis to identify potential targets of shear- and side-dependent miRNA

It is known that miRNAs control gene expression by inversely regulating the expression of mRNAs. This logic was applied to identify the potential mRNA targets of side- and shear- dependent miRNA (s), an in-depth bioinformatics analysis was carried out as outlined in the Fig. 6A. Most of the sequence-based mRNA target databases are available only for 3 species: human, mouse and rat. The porcine miR-214 is homologous with miR-214-3p in human. The experimentally validated mRNA target list of human miR-214-3p, was obtained from the two most popular databases miRWalk^27^ and miRTarBase^26^.

To filter the extensive target list, two additional data sets were used. The first one was the validated side-dependent genes from mRNA microarray data of fresh porcine AV endothelial cells published by Simmons *et al.*^28^. From this data set, the list of mRNA genes (299) that had significantly lower expression in the fibrosa compared to the ventricularis only was considered. The second data set was the mRNA genes list of sheared human AV ECs from our previous experimental data^5^. Since miR-214 is upregulated in fibrosa exposed to OS vs. LS and compared to ventricularis exposed to LS or OS, only mRNA genes that were downregulated in the corresponding data sets were chosen. The number of downregulated genes in these two experimental data sets was 302 and 630 respectively with a false discovery rate of ≤5%. Specifically, the mRNA targets filtered and chosen for further investigation are known to play a role in processes such as cell proliferation, apoptosis, inflammation, endothelial dysfunction, development, and endothelial-to-mesenchymal transition that lead to AV calcification.

### *Ex vivo* Cone and Plate Shear Stress Bioreactor

An *ex vivo* shear stress bioreactor was used to study the effect of shear stresses on AV, especially the expression of miRNAs, in a side-dependent manner. This bioreactor has been designed and extensively validated to expose AV tissues to well-defined shear stress profiles^7^,^51^. In the laboratory, circular 7-mm radius samples were cut aseptically from the basal region of each leaflet under the sterile laminar flow hood. There was an equal distribution of left, right, and non-coronary leaflets for both the control and experimental treatment groups. As detailed by Sucosky *et al.*, each well of the shear stress bioreactor was filled with 1.2% agarose (Thermo Fisher Scientific, NJ), which acted as a tissue holder. The circularly cut tissue samples were placed on top of the agarose bed. Either the fibrosa or ventricularis side was exposed to the shear stress during an experiment. Statically cultured tissues and/or freshly isolated AV leaflets were used as control. To investigate the changes in gene expression (miRNA and mRNA) and valve histology, leaflets were cultured for 2 days using regular DMEM media^52^. We chose a 2 days culture duration, as it is well known that this culture duration is sufficient to induce changes in gene expression and makes it possible to investigate the early onset of AV disease^7^.

For calcification-related studies, leaflets were sheared for 3 and 7 days to determine the time at which detectable calcification occurs. To accelerate the calcification process that occurs over decades *in vivo*, an osteogenic media was used. This osteogenic media contained regular DMEM supplemented with 1 mmol/L glycerophosphate, 10 μ mol/L dexamethasone, 3.8 mmol/L PO_4_^3−^, and 1 ng/ml transforming growth factor (TGF-β 1). This media was previously used to accelerate the calcification of AV leaflets in the presence of cyclic stretch^50^. Media (regular or osteogenic) was changed every 2 days to replenish the nutrients. The entire setup was placed in a 5% CO_2_ incubator at 37 °C. Post-culture, the portion of the tissue unexposed to shear stress was carefully excised (without touching the exposed tissue) using sterile tools under the sterile laminar flow hood. Tissues were briefly rinsed in multiple changes of sterile nuclease-free dPBS maintained at 4 °C until the media was completely removed.

### Shear Stress Conditions

The most common idealized shear stress paradigm is one in which the fibrosa (F) experiences oscillatory shear stress and the ventricularis (V) experiences unidirectional pulsatile shear stress^6^,^7^,^40^. A physiological pulsatile unidirectional shear stress (LS) of 0–79 dyne/cm^2^, with a peak magnitude 79 dyne/cm^2^, was used to investigate the mechanobiology on the ventricularis side. We recently showed that maximum calcification in the fibrosa side was observed when exposed to ± 5 dyne/cm^2^ (low magnitude) compared to ±10 dyne/cm^2^ (physiological) and ± 25 dyne/cm^2^ (high magnitude), within 3 days of culture in osteogenic media^53^. Thus, oscillatory shear stress of low magnitude ± 5 dyne/cm^2^ at 1 Hz (OS) was chosen to study the mechanobiology in the fibrosa side.

For further investigation, samples from four conditions were used: 1) fibrosa exposed to OS, 2) fibrosa exposed to LS, 3) ventricularis exposed to OS, and 4) ventricularis exposed to LS. Fibrosa exposed to OS and ventricularis exposed to LS are physiologically relevant as the sides F/V are exposed to their respective shear stresses. Thus the fibrosa exposed to OS vs. ventricularis exposed to LS comparison indicates side-dependency. Fibrosa exposed to LS and ventricularis exposed to OS are referred to as altered conditions as the physiological shear stress profiles are switched between the two sides. Fibrosa exposed to OS vs. LS and ventricularis exposed to OS vs. LS comparisons were used to compare the shear dependency of the fibrosa and the ventricularis, respectively. Thus, these four shear conditions were used to understand if the preferential calcification in the fibrosa side is a result of its inherent extracellular matrix and cellular composition, or the shear it experiences (OS or LS), or both.

For calcification related studies, experiments were carried out to assess if the calcification induced by this OS required the low magnitude or the oscillatory nature of the shear stress. Then, to determine if the calcification induced by this OS was side-specific, ventricularis was also exposed to OS. Samples were exposed to a steady shear stress of 5 dyne/cm^2^ to test if the calcification induced in the fibrosa side was due to the oscillatory nature or the low magnitude (5 dyne/cm^2^) of the oscillatory shear stress. Although the ventricularis side seldom experiences the shear stress this low, this particular experiment will help us understand how the endothelial cells on ventricularis side will respond to this calcification inducing OS. Further, since the oscillatory shear stress did not induce calcification in the ventricularis side, this control experiment was not done on the ventricularis side.

### Total RNA isolation of sheared AV samples

From 2 day shear experiments, total RNA was isolated from whole tissue (both endothelial and interstitial cells) using the miRNeasy kit (Qiagen, CA). It was technically challenging to isolate endothelial-enriched RNA from the sheared AV samples (also see Supplementary Fig. S1). Hence, it was more practical to isolate total RNA from entire tissue (interstitial and endothelial cells). To achieve sufficient RNA yield for qPCR, three sheared tissues were pooled per isolation. In the case of static culture, it was sufficient to pool just two tissues.

The primer for miR-214 was obtained from Qiagen (CA) and its expression was assessed via qPCR using miScript II RT kit (Qiagen, CA) and miScript SYBR Green PCR kit (Qiagen, CA). RNU6B included in this PCR kit was used as the endogenous control. The primers for mRNA target genes were designed, ordered from Integrated DNA Technologies (CA) and validated using qPCR. These mRNA primer sequences are listed in Supplementary Table S1. The expression of these mRNA targets was also assessed via qPCR using High-Capacity cDNA RT kit with RNAse inhibitor (Life Technologies, NY) and VeriQuest Fast SYBR Green (Affymetrix, CA).

### Analysis of qPCR data

A sample size of at least 3 to a maximum of 12 was used for data analysis. As mentioned earlier, each sample infact represents 2 or 3 tissue samples (due pooling of tissues). The Δ Δ CT method was used to analyze the qPCR data in order to validate the miRNAs identified by the microarray analysis. To analyze qPCR data for all other experiments, Δ CT method was used and fold changes (2^(-Δ CT)) were calculated. When comparing two groups, for instance, fibrosa exposed to OS vs. LS, the fold changes were normalized to the mean of the second group (here fibrosa exposed to LS, which was set as the baseline).

### MiRNA silencing protocol in porcine AV leaflets *ex vivo*

A protocol was developed to deliver anti-miRNA to AV tissue using static culture *ex vivo*. A fluorescently tagged non-targeting (NT) anti-miR (Exiqon, Denmark) was used to optimize the transfection protocol and also to visualize the extent of transfection. AV leaflets were passively transfected with 400 nM tagged NT anti-miR and incubated in OptiMEM for 4 h (Life Technologies, NY). After this, the leaflets were cultured under static conditions for 2 days using regular DMEM. Statically cultured leaflets with no anti-miR were used as a negative control. After 2 days, each leaflet was washed well in nuclease-free sterile dPBS and was cut in half for both RNA isolation and imaging. The leaflet sections used for imaging were counterstained with DAPI and imaged *en face* to determine the transfection efficiency of tagged NT anti-miR. Total RNA was isolated from the other half of each of the leaflets and qPCR analysis was done to determine if the expression of miR-214 was affected by the NT anti-miR transfection.

Since the miR-214 is highly expressed when fibrosa is exposed to OS compared to all other shear conditions, the miR-214 silencing studies were carried out in this shear condition only. The *in vivo* grade non-tagged anti-miR-214 was ordered from Exiqon (Denmark). Following the above protocol, the samples were sheared after 4 h incubation with 400 nM of anti-miR-214 and NT anti-miR for 2 days. After silencing miR-214, the expression of miR-214 and its target mRNAs were determined using qPCR. Experiments with NT anti-miR were used as controls for all comparisons in miRNA silencing studies.

### Functional role of miR-214 in porcine AV leaflets *ex vivo*

In addition to identifying the potential targets regulated by miR-214 in AV, the functional roles of miR-214 in early (endothelial-to-mesenchymal transition) and late (inflammation and calcification) stages of calcific AV disease were also determined. These miR-214 silencing studies with anti-miR-214 were carried out for 2 days, also when fibrosa was exposed to OS. To investigate if silencing miR-214 affects collagen composition, picrosirius red stain was performed. To determine the role of miRNAs in AV calcification, miR-214 silencing experiments were also performed only when fibrosa exposed to OS, for 3 and 7 days (media changed after 2 days) with osteogenic media. Calcium levels were assessed via the Arsenazo III calcium assay. Experiments with NT anti-miR were used as controls for all comparisons in silencing studies.

### Histological assessment of sheared samples

#### Preparation of paraffin or frozen sections

To prepare paraffin sections, the tissues were fixed in 10% neutral buffered formalin (Fisher Scientific, PA) for at least 24 h, saturated in 70% ethanol (VWR, GA), processed in ascending grades of ethanol, embedded in paraffin, and cut into 5 μ m sections. The blocks and the tissue sections were stored at room temperature. The cryopreserved tissue blocks were prepared by embedding the tissue in OCT medium (Electron Microscopy Sciences, PA) and freezing it using liquid nitrogen. Sections with thicknesses of 5 to 7 μ m were cut. The blocks and tissue sections were stored at − 80 °C.

#### Immunofluorescence Staining

To investigate if silencing miR-214 affected the protein expression of its target genes, immunostaining was performed. Another well-known transcription regulator, transforming growth factor beta 1 (TGFβ 1) is highly expressed in calcified AVs and is known to mediate phenotypic switch (fibroblast to myofibroblast), fibrosis, apoptosis, inflammation and calcification. So the possible regulation and activation of TGFβ 1 by miR-214 was also tested. Frozen sections were fixed in a 1:1 mixture of methanol/acetone for 10 min at room temperature (RT) and then blocked (1 h, at RT) using 10% (v/v) donkey serum in PBS. Immunofluorescence staining was carried out using following antibodies, KLF4 (Novus Biologicals, CO), TGFβ 1 at a concentration of (1: 200) overnight at 4 °C. Secondary staining was performed using appropriate secondary antibody labeled with Rhodamine Red X (Invitrogen, NY) for KLF4 or DyLight 488-conjugated donkey anti-rabbit secondary antibody for TGF β 1 (Jackson Immunoresearch, PA) (1:500). Nuclei were counterstained with DAPI (1: 5000) and the samples were mounted using anti-fade mounting medium and imaged. ImageJ software was used to quantify the expression of the proteins in the immunopositive cells as reported by Thayer *et al.*^54^.

#### Verhoeff-Van Gieson Elastin Stain

To visualize the elastin fibers as well as assess the overall thickness of fibrosa, spongiosa and ventricularis layers of the AV ECM, a Verhoeff-Van Gieson elastin stain was performed. AV leaflet samples stained with this elastin stain distinctly show the tri-layered components of the AV ECM: fibrosa, spongiosa and ventricularis layers. Elastin fibers stain blue-black to black, nuclei stains blue to black, collagen stains red, and muscle and other matrix components stain yellow. ACCUSTAIN Elastic kit (Sigma-Aldrich, MO) was used according to the manufacturer’s directions. The thickness of each of these layers was measured using ImageJ software as reported by Thayer *et al.*^54^.

#### Picrosirius Red Collagen Stain

Picrosirius red staining was performed to examine collagen fiber structure and morphology. This stain allows us to distinguish newly formed collagen from mature collagen. Deparaffinized slides were stained in picrosirius red solution (saturated picric acid solution, ready to use, Sigma-Aldrich, MO) for one hour. The slides were then washed in 0.5% acidified water (5 mL glacial acetic acid in 995 mL distilled water), and rinsed in three changes of 100% ethanol, and xylene before being mounted in a resinous medium and coverslipped. When viewed under a bright-field microscope collagen appears red on a pale yellow background. When examined through a circularly polarized filter the larger (or mature) collagen fibers appear bright red or orange, thin (or young) fibers are yellow, and thinner fibers (or nascent) are green. This principle was used to quantify the relative proportion of the collagen fibers in the sheared AV leaflets (depending on their maturity) using a custom Matlab (The Mathworks, Natick, MA) code^52^. Since the red and orange correspond to the mature collagen, their percentages were combined for analysis in this study.

### Calcium assay and mineralization stain

Tissue samples from calcification experiments were pulverized by mortar and pestle in liquid nitrogen, and collected in pre-weighed vials. After sample collection, the vials were re-weighed before and after drying the samples overnight at 37 °C. The dry weights of the tissue were then computed. Subsequently, the ground samples were incubated in 1 M acetic acid at 4 °C for 24 h, to solubilize calcium. The samples were then centrifuged at 15,000 rpm for 9 min, and the supernatant was collected. The supernatant as well as calcium standards were assayed for calcium content by using a calcium specific Arsenazo dye reagent (Fisher-Scientific, MA): 25 μ L of the supernatant or the calcium standard was mixed with 300 μ L of Arsenazo solution in a 96 well plate. The absorbance of the various samples at a wavelength of 650 nm was measured through spectrophotometry, using a 96 well plate reader (BioTek, VT). The absorption calibration curve was established with the calcium standard spectrophotometry readings, and was thereafter applied to readings from the samples. The amount of calcium per dry weight of sample tissue was then calculated.

Von Kossa staining was performed to visualize tissue mineralization. Briefly, frozen sections were allowed to warm to room temperature, and were hydrated in PBS for 2 min. They were then incubated in 1% silver nitrate solution in a clear glass coplin jar placed under ultraviolet light for 2 h. After washing in several changes of distilled water, excess silver nitrate was removed by incubating in 5% sodium thiosulfate for 5 min. The slides were rinsed in distilled water, dehydrated through graded alcohol and cleared in xylene before being mounted in a resinous medium and coverslipped. Calcium deposits appeared black when viewed under normal white light.

### Cell Apoptosis Stain

A cell apoptosis stain was performed to assess the cell death and viability of samples from calcification and miRNA silencing studies. This staining was done using the *in situ* Cell Death Detection Kit; TMR red, TUNEL kit (Roche Diagnostics, Germany). Cryopreserved tissue sections were fixed with 4% paraformaldehyde (Sigma-Aldrich, MO) in dPBS (pH 7.4) for 20 min, rinsed with dPBS for 30 min, permeabilized with 0.1% Triton X-100 (Sigma-Aldrich, MO) in dPBS (2 min at 4 °C), and rinsed twice with dPBS. Staining was performed by incubating tissue sections for 1 h at 37 °C in a humidified chamber in the dark in 50 μ L of TUNEL reaction mixture followed by counterstaining with DAPI for 5 min. A positive control was prepared by incubating the fixed and permeabilized tissue section with DNAse-I solution (3–3000 U/ml RNAse free DNAse-I (Qiagen, CA) in 50 mM TrisCl (Sigma-Aldrich, MO), pH 7.5, 1 mg/mL BSA (Fisher Scientific, PA) for 10 min prior to labeling with TUNEL reaction mixture. A negative control was prepared by incubating fixed and permeabilized tissue sections in 50 μ l of TUNEL label solution (without terminal transferase). Apoptotic cells appeared red and nuclei appeared blue under fluorescence microscopy.

### Imaging

A Zeiss Axioscope A1 (Germany) was used for brightfield imaging. A circular polarizer and analyzer and a rotating stage was used with this microscope to image the tissue sections stained with picrosirius red. Confocal microscopy for miRNA silencing experiments was performed using a 710 NLO laser-scanning microscope (Carl Zeiss, Germany). Immunofluorescence stained sections were imaged using a Zeiss LSM 510 META confocal microscope (Carl Zeiss, Germany).

### Statistical analysis

Data was expressed as mean ±  standard error. All the statistical analyses were done using SPSS Statistics for Mac (Version 20.0, IBM Corp, NY). The normality of all the data was tested using the Anderson-Darling method. Student t-test was used when only two groups were being compared. Two-way ANOVA was used for analyzing independent sample sets with Tukey’s post-hoc test for comparisons between multiple groups. If the data was not normally distributed, Mann-Whitney and Friedman tests were used in place of the student t-tests and ANOVA, respectively. The groups were considered to be significantly different or show trends if the p-value was less than or equal to 0.05 and 0.10, respectively.

### Limitations

The *ex vivo* shear studies provide a critical experimental platform to determine the role of shear in regulating gene expression in a side-dependent manner, while excluding numerous other complex systemic changes occurring *in vivo*. Also, it provides an experimental system that is difficult to achieve in *in vitro* systems such as native ECM and valvular interstitial cells. However, some limitations to this study exist. Due to lack of availability of healthy human valves, healthy porcine valves were used for this research. Endothelial-enriched RNA was used for the microarray experiments, while it was technically challenging to obtain endothelial-enriched RNA from sheared AV leaflets. Hence, RNA from whole AV tissue was used in this case. Further, when the microarray studies were carried out, the human based microarray was used because a porcine specific microarray was not available. However, since the side-dependent miRNAs identified in this study are conserved in humans, investigating the functional role of these miRNAs in porcine AV leaflets will still provide us clues about their role in AV inflammation and calcification in humans. Thus, despite these limitations, our data show the possibility of miRNA involvement in side-dependent AV disease.

## Additional Information

**How to cite this article**: Rathan, S. *et al.* Identification of side- and shear-dependent microRNAs regulating porcine aortic valve pathogenesis. *Sci. Rep.* **6**, 25397; doi: 10.1038/srep25397 (2016).

## Supplementary Material

Supplementary Information
