# Supplementary material for: Identification of side- and shear-dependent microRNAs regulating porcine aortic valve pathogenesis
Source: Sci Rep. 2016 May 6;6:25397. doi: 10.1038/srep25397 (PMC4858741; doi:10.1038/srep25397)
Supplement: Supplementary Information [file srep25397-s1.doc]

**Identification of side- and shear-dependent microRNAs regulating porcine aortic valve pathogenesis**

Supplementary document

Swetha Rathan^1^, Casey J. Ankeny^2^, Sivakkumar Arjunon^3^, Zannatul Ferdous^4^, Sandeep Kumar^3^, Joan Fernandez Esmerats^3^, Jack M. Heath^3^, Robert M. Nerem^5^, Ajit P. Yoganathan^1, 3*^ and Hanjoong Jo^3*^

1. School of Chemical and Biomolecular Engineering, Georgia Institute of Technology, Atlanta, GA
2. School of Biological and Health Systems Engineering, Arizona State University, Tempe, AZ
3. The Wallace H. Coulter Department of Biomedical Engineering, Georgia Institute of Technology and Emory University, Atlanta, GA
4. Mechanical, Aerospace and Biomedical Engineering, University of Tennessee
   Knoxville, TN
5. Parker H. Petit Institute for Bioengineering and Bioscience, Georgia Institute of Technology, Atlanta, GA

* Ajit P. Yoganathan and Hanjoong Jo are co-corresponding authors.

Address for correspondence and reprints:

Ajit P. Yoganathan, Ph.D*

The Wallace H. Coulter Department of Biomedical Engineering

Georgia Institute of Technology and Emory University

BME-TEP, 387 Technology Circle NW, Suit 232

Atlanta, GA 30313

Tel: 404.894.2849, Fax: 404.385.1268

E-mail: ajit.yoganathan@bme.gatech.edu

**Supplementary Results**


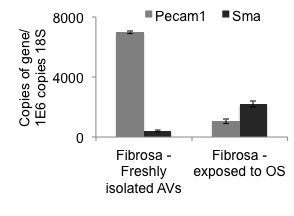


**Supplementary Figure S1.** The RNA isolated from the endothelial layer of fibrosa of freshly isolated AVs was endothelial-enriched (high expression of *Pecam1* and low expression of *Sma*). However we were unable to isolate endothelial-enriched RNA from the endothelial layer of sheared fibrosa (fibrosa exposed to OS). In this case, the expression of *Pecam1* was low where as the expression of *Sma* was high. n=4.

**Supplementary Figure S2.** Positive control for TUNEL showed excessive apoptosis (red). Negative control for TUNEL showed no signs of apoptosis. No apoptotic cells were observed in freshly isolated AVs, and when fibrosa or ventricularis was exposed to OS for 3 days in osteogenic media. However, a few apoptotic cells were observed (arrows point to red tagged nuclei) statically cultured tissues were treated with NT anti-miR. F: fibrosa, V: ventricularis.

**Supplemental Figure S3**. A) Only *Enos* showed a lower expression trend in fibrosa compared to ventricularis when exposed to OS. B) No changes were observed in gene expression in ventricularis in a shear-dependent manner (OS vs. LS). n=3 (pooled 3 samples/isolation), #p≤0.1.


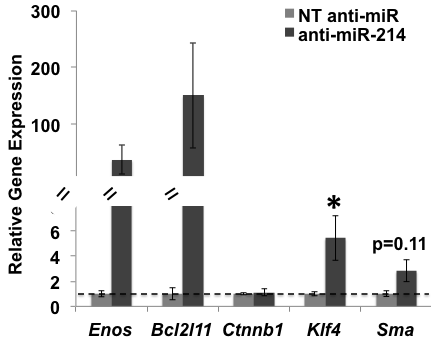


**Supplemental Figure S4.** Out of 5 selected mRNA targets, silencing of miR-214 in fibrosa when exposed to OS significantly upregulated *Klf4* (*p=0.05). The *Sma* showed an increasing trend that did not reach statistical significance. n=6-8 (pooled 3 samples/isolation).

Supplementary Table S1. Validated mRNA primer sequences used in this study

| **Gene** | **Forward Primer** | **Reverse Primer** |
| --- | --- | --- |
| *Enos* | 5- GAG ACT GGC CTT ATT CCT GGG -3 | 5- CTC CGG AAT ACC ACT GCT GG -3 |
| *Bcl2l11* | 5-TGA GTC GGA TCG CAA CTT GG-3 | 5-ATC GGT TGA AGC GTT CCT GG-3 |
| *Ctnnb1* | 5-GGA CAT CAC TGA ACC TGC CA-3 | 5-GGT AGT CCG TAG TGA AGG CG-3 |
| *Col1a1* | 5-GGC CAA GAA GAA GAC ATC CCA-3 | 5-ACA ACA CAT TGC CGT TGT CG-3 |
| *Klf4* | 5-CTA AGC AGC AGG GAC TGT CA-3 | 5-GTG GCA TGA GCT CTT GGT AAT G-3 |
| *Sma* | 5- GGT GGG AAT GGG ACA AAA AGA C -3 | 5- TGG TGA TGA TGC CGT GTT CTA TT -3 |
| *18S* | 5-CCC CAA CTT CTT AGA GGG ACA AG-3 | 5-AGG GCA TCA CAG ACC TGT TAT TG-3 |
| *Pecam1* | 5- ATC TGC ATC TCG TGG GAA GT -3 | 5- GAG CTG AAG TGT CAG CAG GA -3 |

**Supplementary Data**

| **Side-specific miRNAs in Porcine AV Endothelium** | | | | |
| --- | --- | --- | --- | --- |
|  |  |  |  |  |
| **Gene Name** |  | **Fold Change** |  | **q-value(%)** |
| ggo-miR-199a_st |  | 2.122027962 |  | 0 |
| gga-miR-199_st |  | 2.173988035 |  | 0 |
| dre-miR-199_st |  | 2.458473265 |  | 0 |
| hsa-miR-181a_st |  | 2.326768482 |  | 0 |
| fru-miR-199_st |  | 2.055957345 |  | 0 |
| hsa-miR-199a-5p_st |  | 2.061154999 |  | 0 |
| mml-miR-199a-5p_st |  | 1.974896436 |  | 0 |
| sla-miR-199a_st |  | 2.254336167 |  | 0 |
| sla-miR-181a_st |  | 1.99746792 |  | 0 |
| mmu-miR-181a_st |  | 2.659618528 |  | 0 |
| xtr-miR-181a_st |  | 2.143493437 |  | 0 |
| tni-miR-199_st |  | 2.735916121 |  | 0 |
| ptr-miR-199a_st |  | 2.745057432 |  | 0 |
| rno-miR-199a-5p_st |  | 2.032199447 |  | 0 |
| mml-miR-199a_st |  | 2.033437995 |  | 0 |
| fru-miR-181a_st |  | 1.870696263 |  | 0 |
| mne-miR-199a_st |  | 2.299408835 |  | 0 |
| dre-miR-100_st |  | 1.804606558 |  | 0 |
| bta-miR-199a-5p_st |  | 2.545981613 |  | 0 |
| mdo-miR-181b_st |  | 2.394053955 |  | 1.490638278 |
| rno-miR-181a_st |  | 1.997827933 |  | 1.490638278 |
| cfa-miR-199_st |  | 2.87447138 |  | 1.490638278 |
| ptr-miR-181a_st |  | 2.316335666 |  | 1.490638278 |
| hsa-miR-214_st |  | 1.854785474 |  | 1.490638278 |
| ppa-miR-199a_st |  | 2.291207309 |  | 1.490638278 |
| lla-miR-181a_st |  | 2.568861305 |  | 1.490638278 |
| dre-miR-181a_st |  | 1.74890792 |  | 1.490638278 |
| gga-miR-181a_st |  | 1.908520478 |  | 1.490638278 |
| age-miR-100_st |  | 1.64354239 |  | 1.490638278 |
| bta-miR-181a_st |  | 1.827608849 |  | 1.490638278 |
| mmu-miR-199a-5p_st |  | 3.492210848 |  | 1.490638278 |
| rno-miR-181b_st |  | 1.766611735 |  | 1.490638278 |
| rno-miR-199a-3p_st |  | 4.228381848 |  | 1.490638278 |
| xtr-miR-199a_st |  | 1.987012112 |  | 2.235957417 |
| ggo-miR-100_st |  | 1.810186906 |  | 2.235957417 |
| mmu-miR-199b_st |  | 3.23397382 |  | 2.235957417 |
| mmu-miR-100_st |  | 1.691903948 |  | 2.235957417 |
| ggo-miR-130a_st |  | 1.565110661 |  | 2.235957417 |
| ggo-miR-181b_st |  | 2.515420046 |  | 2.235957417 |
| mne-miR-181a_st |  | 1.895753868 |  | 2.235957417 |
| ssc-miR-214_st |  | 1.633428532 |  | 2.235957417 |
| xtr-miR-199a-star_st |  | 3.21541585 |  | 2.235957417 |
| mml-miR-199a-3p_st |  | 3.21410041 |  | 2.235957417 |
| ppa-miR-100_st |  | 1.653590112 |  | 2.235957417 |
| mdo-miR-181c_st |  | 1.951881768 |  | 2.235957417 |
| hsa-miR-100_st |  | 1.638841881 |  | 2.235957417 |
| xtr-miR-181b_st |  | 2.399651113 |  | 2.235957417 |
| fru-miR-100_st |  | 1.684783641 |  | 2.235957417 |
| bta-miR-199a-3p_st |  | 3.298447937 |  | 2.235957417 |
| lla-miR-199a_st |  | 2.06491825 |  | 2.235957417 |
| lla-miR-100_st |  | 1.627392973 |  | 2.235957417 |
| bta-miR-181b_st |  | 2.435168609 |  | 2.235957417 |
| mml-miR-181a_st |  | 1.649972142 |  | 2.235957417 |
| ptr-miR-100_st |  | 1.650609673 |  | 2.235957417 |
| rno-miR-25_st |  | 2.051565193 |  | 2.235957417 |
| cfa-miR-181a_st |  | 3.108918937 |  | 2.235957417 |
| mdo-miR-100_st |  | 1.888295216 |  | 2.235957417 |
| mne-miR-127_st |  | 1.846570913 |  | 2.235957417 |
| gga-miR-181b_st |  | 2.169131726 |  | 2.235957417 |
| dre-miR-181c_st |  | 2.254511705 |  | 2.235957417 |
| ppa-miR-181a_st |  | 2.325091781 |  | 2.235957417 |
| dre-miR-199-star_st |  | 3.230966965 |  | 2.235957417 |
| dre-miR-25_st |  | 1.607698638 |  | 2.235957417 |
| hsa-miR-199b-3p_st |  | 3.417698977 |  | 2.235957417 |
| tni-miR-181a_st |  | 1.825556095 |  | 2.235957417 |
| hsa-miR-181b_st |  | 2.146019224 |  | 2.235957417 |
| ggo-miR-181a_st |  | 1.832556484 |  | 2.851655836 |
| mml-miR-150_st |  | 1.541112662 |  | 2.851655836 |
| tni-miR-100_st |  | 1.564537603 |  | 2.851655836 |
| lla-miR-181b_st |  | 2.31963691 |  | 3.988464582 |
| hsa-miR-708_st |  | 1.901627054 |  | 3.988464582 |
| mmu-miR-532-5p_st |  | 1.690061798 |  | 3.988464582 |
| xtr-miR-100_st |  | 1.600960079 |  | 3.988464582 |
| mdo-miR-214_st |  | 1.702098664 |  | 3.988464582 |
| mmu-miR-199a-3p_st |  | 3.080342276 |  | 4.591165896 |
| osa-miR809b_st |  | 1.674776601 |  | 5.825257481 |
| ppy-miR-199a_st |  | 1.949026897 |  | 8.998365215 |
| mne-miR-181b_st |  | 1.573219165 |  | 8.998365215 |
| rno-miR-100_st |  | 1.488521094 |  | 8.998365215 |
| mml-miR-181b_st |  | 1.926562069 |  | 8.998365215 |
| hsa-miR-150_st |  | 2.029511713 |  | 8.998365215 |
| hsa-miR-199a-3p_st |  | 3.410530899 |  | 8.998365215 |
| ggo-miR-214_st |  | 1.780276507 |  | 8.998365215 |
| mmu-miR-181d_st |  | 1.782840534 |  | 9.955334214 |
| ppa-miR-181b_st |  | 2.712655173 |  | 9.955334214 |
| mml-miR-25_st |  | 2.295695029 |  | 11.8737049 |
| cfa-miR-708_st |  | 2.013370945 |  | 11.8737049 |
| xtr-miR-25_st |  | 2.198123329 |  | 12.71229722 |
| ssc-miR-181b_st |  | 2.338986714 |  | 12.71229722 |
| cfa-miR-369_st |  | 1.500068796 |  | 12.71229722 |
| dre-miR-181b_st |  | 1.879045064 |  | 13.75234024 |
| ppy-miR-100_st |  | 1.46731471 |  | 13.75234024 |
| ssc-miR-181c_st |  | 1.623301522 |  | 13.75234024 |
| mml-miR-100_st |  | 1.538016165 |  | 13.75234024 |
| ppy-miR-181a_st |  | 1.816016948 |  | 13.75234024 |
| hsa-miR-455-3p_st |  | 2.04663902 |  | 14.75731895 |
| gga-miR-199-star_st |  | 2.005085838 |  | 14.75731895 |
| ame-miR-100_st |  | 1.49954034 |  | 14.75731895 |
| fru-miR-25_st |  | 2.060147071 |  | 14.75731895 |
| hsa-miR-618_st |  | 1.513205229 |  | 14.75731895 |
| rno-miR-214_st |  | 1.608209473 |  | 14.75731895 |
| ppa-miR-25_st |  | 1.847027056 |  | 16.71540982 |
| xtr-miR-130a_st |  | 1.439070894 |  | 16.71540982 |
| aga-miR-100_st |  | 1.506975888 |  | 17.02767571 |
| sla-miR-100_st |  | 1.659381952 |  | 20.88299852 |
| mdo-miR-181a_st |  | 2.408092817 |  | 20.88299852 |
| cfa-miR-543_st |  | 1.40061101 |  | 20.95174913 |
| hsa-miR-523-star_st |  | 1.69790609 |  | 20.95174913 |
| mmu-miR-708_st |  | 1.675250688 |  | 20.95174913 |
| mmu-miR-181c_st |  | 1.715009617 |  | 20.95174913 |
| tni-miR-181b_st |  | 2.024126328 |  | 20.95174913 |
| hsa-miR-223_st |  | 1.532971986 |  | 20.95174913 |
| osa-miR395s_st |  | 1.430450261 |  | 28.28486133 |
| mne-miR-214_st |  | 1.849738505 |  | 28.28486133 |
| mdo-miR-199b_st |  | 1.405798675 |  | 28.28486133 |
| ppt-miR166k_st |  | 1.544284613 |  | 28.28486133 |
| fru-miR-181b_st |  | 1.778519345 |  | 28.28486133 |
| ppy-miR-214_st |  | 1.456885335 |  | 28.28486133 |
| cfa-miR-25_st |  | 2.219899966 |  | 28.28486133 |
